# When brakes fail you: Oculocardiac reflex elicited by a retained foreign body in a penetrating orbital injury

**DOI:** 10.1016/j.ajoc.2024.102029

**Published:** 2024-02-29

**Authors:** Sabrina Abu Hassan Asaari, Dharshini Balasubramaniam, Norlina Ramli, Fazliana Ismail

**Affiliations:** Universiti Malaya Eye Research Centre, Department of Ophthalmology, Universiti Malaya, Kuala Lumpur, Malaysia

**Keywords:** Oculocardiac reflex, Penetrating orbital injury, Intraorbital foreign body, Orbital floor fracture

## Abstract

**Purpose:**

To report a case of oculocardiac reflex following penetrating orbital injury with entrapment of extraocular muscle secondary to a retained orbital foreign body.

**Observation:**

A 19-year-old man with no known comorbidities presented with a foreign object in his right orbit following a motor vehicle accident. Visual acuity was 20/20 bilaterally with positive relative afferent pupillary defect for the right eye. A motorcycle brake lever was embedded in the right inferotemporal conjunctival fornix, missing his globe. He was bradycardic in the emergency department, with a pulse rate ranging between 45 and 48 beats per minute. An urgent computed tomography scan of the orbit confirmed the penetrating injury with a linear hyperdense foreign body extending from the right inferior orbit into the right maxillary sinus. This foreign body was seen abutting the right lateral rectus and the globe inferiorly. Fractures involving the inferior and medial wall of the right orbit were seen with the inferior rectus, and orbital fat herniated into the maxillary sinus.

The patient underwent urgent orbit exploration with foreign body removal and orbital floor repair under general anesthesia. Immediately after removing the foreign body, his pulse rate returned to normal, within 72–80 beats per minute. Six months postoperatively, visual acuity was 20/20 for both eyes. Although he had persistent diplopia on upgaze, he refused any other interventions.

**Conclusion and importance:**

Prompt detection of the oculocardiac reflex and removal of the inciting stimulus is vital to prevent any life-threatening events.

## 1 Introduction

The Oculocardiac reflex (OCR) is a vagal response elicited when extraocular muscle traction leads to bradycardia, arrhythmias and even hemodynamic shock. In trauma patients, OCR is reported to occur in 17% of orbital blowout fractures.^1^ These cases are related to increased muscle tension from direct muscular entrapment, indirect entrapment of adjacent orbital fat or retained intraorbital foreign bodies. The presenting clinical features may develop acutely or delayed. Here we report a case of OCR elicited by an entrapment of extraocular muscle from a retained orbital foreign body.

## 2 Case report

**Fig. 1:**
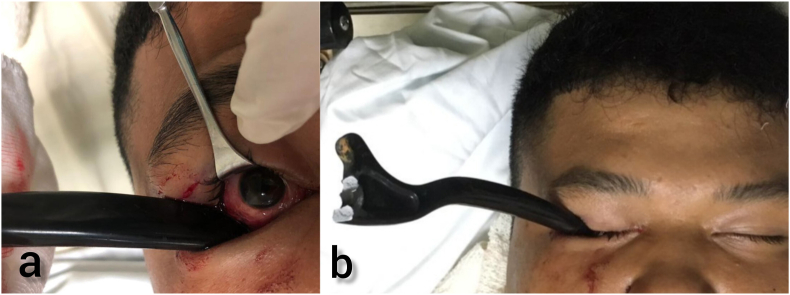
Metallic motorcycle brake lever entering inferior orbit via the inferotemporal conjunctival fornix.

A 19-year-old man with no known medical comorbidities was brought to the emergency department by an ambulance after a motor vehicle accident. Paramedics were informed that the motorcycle brake lever had to be cut by the fire brigade at the accident scene as it had embedded into the patient's right eye socket. On examination, a metallic motorcycle brake lever was seen embedded in the right inferior orbit via the inferotemporal conjunctival fornix (Fig. 1(a and b)). His visual acuity was 20/20 bilaterally with a positive relative afferent pupillary defect in the right eye. The anterior segment was unremarkable. Dilated fundus examination revealed a pink disc with inferior commotio retinae. The globe was intact, and no intraocular foreign bodies were detected.

**Fig. 2:**
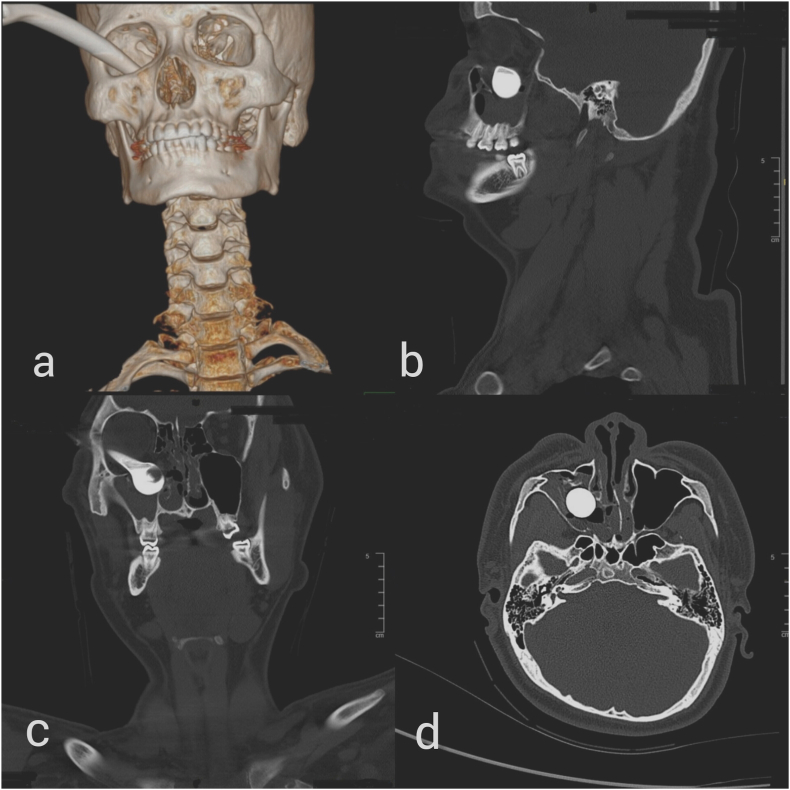
(a) CT imaging with 3D reconstruction showing entry of brake lever into orbit. CT imaging in (b) sagittal cut, (c) coronal cut and (d) axial cut showing the bulbous tip of the brake within the maxillary sinus.

The patient was noted to be constantly bradycardic with a pulse rate between 45 and 48 beats per minute and a junctional rhythm was detected on electrocardiogram. It was also noted that manipulation of the foreign body resulted in exacerbation of the bradycardia. His Glasgow coma scale was full, and other vitals were normal. An urgent computed tomography of the orbit confirmed the penetrating orbital injury with a linear hyperdense foreign body extending from the right inferior orbit into the right maxillary sinus. This foreign body was seen abutting the right lateral rectus and the globe inferiorly. The globe was displaced superiorly. There was a fracture of the inferior wall of the right orbit, right lamina papyracea, medial wall of the right maxilla and vomer. The right inferior rectus and orbital fat herniated inferiorly into the maxillary sinus (Fig. 2(a–d)).

**Fig. 3:**
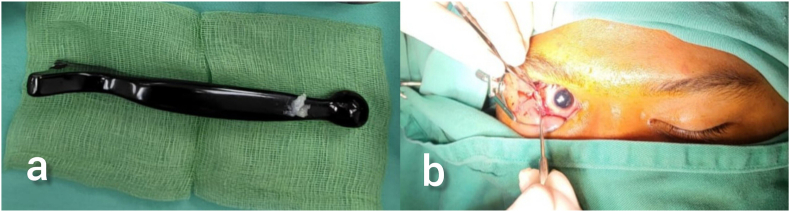
(a) Metallic brake handle measuring 17cm in length. (b) Inferotemporal conjunctival fornix entry point.

At this point, OCR was diagnosed, and the patient underwent urgent orbit exploration with removal of foreign body and orbital floor repair under general anesthesia together with the oral maxillofacial team (Fig. 3(a and b)).

On the table, a gentle forced duction test was performed showing restriction in all gazes. Once the foreign body was removed, his pulse rate immediately returned to normal range within 72–80 beats per minute. A lower lid laceration involving the lid margin and lateral canthus was also repaired. Exploration confirmed that the inferior oblique, lateral and inferior recti muscles were intact and absence of globe rupture. The orbital floor was reconstructed using a pre-formed orbital floor plate and fixed with 2 screws. Medial wall fracture was not repaired as it was small. Post orbital floor repair, a repeat forced duction test was performed, verifying the freedom of all extraocular muscles.

**Fig. 4:**
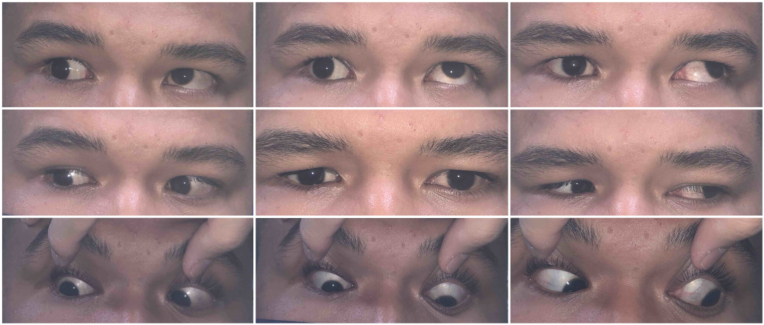
9 cardinal positions of gaze show limited elevation of the right eye.

Six months postoperatively, visual acuity was 20/20 for both eyes with resolved right inferior commotio retinae. There was no diplopia on primary and downgaze. The right eye appeared slightly enophthalmic with 5mm difference on exophthalmometer with persistent diplopia on upgaze (Fig. 4). However, he refused any further interventions.

## 3 Discussion

The Oculocardiac reflex (OCR), also known as the Aschner reflex or trigeminovagal reflex is a physiologic response to physical stimulation of the extraocular muscles and ocular adnexa. The afferent limb is mediated via the ophthalmic division of the trigeminal nerve. The efferent limb is via the vagus nerve, which is responsible for decreasing the activity of the sinoatrial node, thus reducing pulse rate. This neuroprotective action physiologically aids in conserving oxygen by inducing relative bradycardia and lowering blood pressure.^1^^,^^2^ Clinical features range from asymptomatic or mild vasovagal symptoms to rarely asystole.^3^, ^4^, ^5^, ^6^

OCR occurs in association with orbital trauma via a multitude of mechanisms. These include muscle and fat entrapment or globe displacement in orbital floor fractures, tissue manipulation during assessment or surgical repair and intraorbital foreign bodies causing pressure onto orbital structures.^5^, ^6^, ^7^, ^8^ In this case, we believe the OCR was elicited by the entrapment of the inferior rectus muscle on the retained orbital foreign body through the orbital floor and medial wall fractures. Only a few case reports of OCR elicited by foreign body have been published to date. Singh et al. reported a case of a young child with a wooden intraorbital foreign body who developed cardiogenic shock.^7^ Similarly, Yilmaz et al. described a gunshot wound patient with a metallic intraorbital foreign body who required a pacemaker insertion.^6^

Bradycardia may exhibit late, even years after evisceration.^5^^,^^6^ This may be due to manipulation of the intraorbital contents or retained foreign bodies. Greater strength and duration of the stimulus may increase the incidence of OCR.^1^^,^^9^^,^^10^ Although tension of any muscle can elicit OCR, it is more commonly seen in inferior or medial recti muscles.^1^^,^^2^ Authors postulate that blowout fractures more frequently entrap inferior recti muscles, whereas the thinness of the medial orbital wall leads to prolapse of the medial rectus instead.^2^ The pediatric age group is at a higher risk of OCR due to greater baseline pulse rates and variability and greater elasticity of the orbital rim leading to more trapdoor-type fractures.^4^^,^^11^

There is extensive data on the occurrence of OCR during surgical procedures.^1^^,^^9^^,^^10^ Ophthalmic surgeries have the highest risk, followed by base of skull and other craniofacial surgeries.^9^^,^^10^ Other risk factors that need to be identified include a background of cardiac disease, using beta-blockers or calcium channel blockers, light general anesthesia, hypercapnia, or hypoxemia.^1^^,^^9^^,^^10^

Patients with polytrauma or concomitant injuries pose diagnostic dilemmas. Bradycardia with hypertension may elude intracranial hypertension, whereas a case of bradycardia with hypotension may indicate hemorrhage, emboli, cardiac tamponade, or pneumothorax.^10^^,^^11^ A high index of suspicion, identification of risk factors and prompt diagnosis are crucial to avoid further deterioration as in our case. Secondary complications of OCR can be difficult and complex to manage. Pham et al. describe a 40-year-old who required intubation for autonomic shock after a total blowout orbital fracture with dislocation of the globe and recti into the maxillary sinus.^8^

Recommendations for the timing of surgical intervention in orbital wall fractures or intraorbital foreign body removal vary.^1^^,^^13^, ^14^, ^15^ Intraorbital foreign bodies necessitate urgent removal if they are organic in material or if secondary complications arise.^12^, ^13^ If OCR is present, removing the stimuli becomes paramount.^2^ Priority for treatment is first to preserve life, second to preserve vision, and finally to preserve the globe. If OCR is present, cardiac monitoring should be initiated. The approach for surgical removal is tailored based on the patient's injuries.^1^ Subconjunctival incision with lateral canthotomy are reported to have better cosmetic outcomes.^2^ However, ensuring adequate exposure for deep exploration should take precedence. Immediate relief in compression to the globe and muscle tension should terminate the reflex, such as in this case. Intravenous anticholinergics such as atropine or glycopyrrolate must be available throughout the procedure.^1^^,^^9^

In our case, the patient is fortunate to have sustained an extensive injury that narrowly missed damaging the globe and intraocular contents, ultimately preserving his vision.

## 4 Conclusion

Prompt detection of the oculocardiac reflex and removal of the inciting stimulus is vital to prevent any life-threatening events.

## Patient consent

Patient consented to publication of the case and audiovisual information.

## Funding

No funding or grant support.

## Authorship

All authors attest they meet the current ICMJE criteria for Authorship.

## CRediT authorship contribution statement

**Sabrina Abu Hassan Asaari:** Writing – original draft. **Dharshini Balasubramaniam:** Data curation. **Norlina Ramli:** Data curation. **Fazliana Ismail:** Writing – review & editing, Data curation.

## Declaration of competing interest

The authors declare that they have no known competing financial interests or personal relationships that could have appeared to influence the work reported in this paper.
